# Glucose Levels of the Oral Glucose Tolerance Test (oGTT) Can Predict Adverse Pregnancy Outcomes in Women with Gestational Diabetes (GDM)

**DOI:** 10.3390/jcm12113709

**Published:** 2023-05-27

**Authors:** Selina Balke, Petra Weid, Laura Fangmann, Paul Rostin, Wolfgang Henrich, Josefine Theresia Koenigbauer

**Affiliations:** Department of Obstetrics, Charité-Universitätsmedizin, 10117 Berlin, Germany; petra.weid@charite.de (P.W.); laura.c.fangmann@gmail.com (L.F.); paul.rostin@charite.de (P.R.); wolfgang.henrich@charite.de (W.H.); josefine.koenigbauer@charite.de (J.T.K.)

**Keywords:** oGTT, oral glucose tolerance test, gestational diabetes, GDM, cesarean section

## Abstract

Objectives and Background: Gestational diabetes (GDM) is a common pregnancy complication defined as a glucose intolerance diagnosis during pregnancy. GDM is strongly associated with adverse fetal and maternal outcomes. In Germany, to screen and diagnose GDM we use a 1 h 50 g oGCT (oral glucose challenge test) followed by a 2 h 75 g oGTT if the first was pathological. This analysis examines the correlation of 75 g oGTT glucose levels and fetomaternal outcome. Methods: Data from 1664 patients from a gestational diabetes consultation clinic at the Charité University Hospital in Berlin, Germany, were analyzed retrospectively from 2015 to 2022. The 75 g oGTT blood glucose levels were categorized into isolated fasting hyperglycemia (GDM-IFH), isolated post-load hyperglycemia (GDM-IPH) and combined hyperglycemia (GDM-CH), using the levels of the fasting, 1 h and 2 h values, after glucose application. These subtypes were compared based on their baseline characteristics as well as fetal and maternal outcome. Results: GDM-IFH and GDM-CH women displayed higher pre-conceptional BMI and required insulin therapy more frequently (*p* < 0.001). The GDM-IFH group was at higher risk of having a primary cesarean section (*p* = 0.047), while GDM-IPH women were significantly more likely to have an emergent cesarean section (*p* = 0.013). The offspring of GDM-IFH and GDM-CH women were born with a significantly higher mean birthweight (*p* < 0.001) and birth weight percentiles (*p* < 0.001) and were at increased risk of being large for gestational age (LGA) (*p* = 0.004). Women from the GDM-IPH group delivered significantly more neonates who were small for gestational age (*p* = 0.027) or with low fetal weight <30th percentile (*p* = 0.003). Conclusion: This analysis shows a strong association between the glucose response pattern in the 75 g oGTT and adverse perinatal fetomaternal outcome. The differences among the subgroups, specifically concerning insulin therapy, mode of delivery and fetal growth, suggest an individualized approach to prenatal care after a GDM diagnosis.

## 1. Introduction

**Figure 1:**
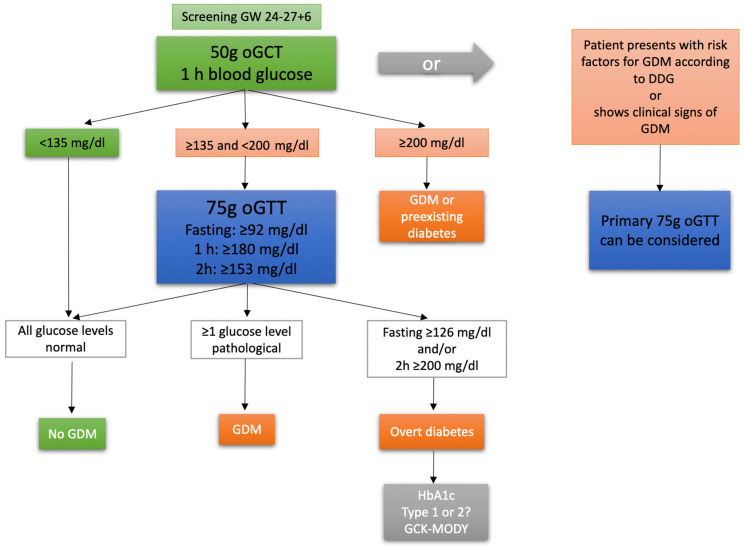
Screening and diagnostic process of GDM in Germany. Initially a 50 g oral glucose challenge test (oGCT) is offered to every pregnant woman between 24 and 28 gestational weeks (GW). Depending on the blood glucose level, further testing for GDM is required. In women with risk factors for GDM according to the Deutsche Diabetes Gesellschaft (DDG) or signs of GDM, a 75 g oral glucose tolerance test (oGTT) as a first-line diagnostic test is possible.

Gestational diabetes mellitus (GDM) is a common complication during pregnancy. In Germany, the incidence of GDM rose from 4.6% of all hospital deliveries in 2013 to 6.8% in 2018 [1]. GDM is defined as an impairment of glucose tolerance that has been diagnosed for the first time during pregnancy [2]. With its prevalence rising, and its well-known associations with other various pregnancy complications such as pre-eclampsia, cesarean section (CS), macrosomia, shoulder dystocia, childbirth injury, postpartum hemorrhage or premature birth, understanding GDM fully is key to improve prenatal care and minimize the risks for mother and child [3,4,5,6,7]. GDM is still, generally, treated as a homogenous disease during pregnancy, although research indicates that a more differentiated approach might be needed, as phenotypical subtypes of the condition seem to be associated with different perinatal outcomes. A possible approach that has been suggested is to focus on the extent of insulin sensitivity and insulin secretion impairment or to differentiate between the two [8,9,10,11]. Another, possibly more practicable, approach is to differentiate GDM subtypes based on the glucose levels observed in the three-point 75 g oral glucose tolerance test (oGTT), which is conducted in the late second to early third trimester of pregnancy and comprises fasting blood glucose measurements, one and two hours after the ingestion of a 75 g glucose solution (Figure 1) [2]. These measurements are widely available through the prenatal file and are, therefore, easy to access. The HAPO study demonstrated an association of maternal plasma glucose levels with large for gestational age (LGA) offspring, primary CS, shoulder dystocia or birth injury, pre-eclampsia and other adverse outcomes [12]. Therefore, the correlation of 75 g oGTT glucose values and fetomaternal outcome has been the subject of several studies [5,13,14,15]. The aim of our study is to corroborate and add evidence to the recent findings by assessing the characteristics of GDM subtypes (isolated fasting hyperglycemia = GDM-IFH, isolated post-load hyperglycemia = GDM-IPH, combined hyperglycemia = CH based on oGTT glucose values) and their respective risk of adverse maternal and fetal outcomes.

## 2. Materials and Methods

**Figure 2:**
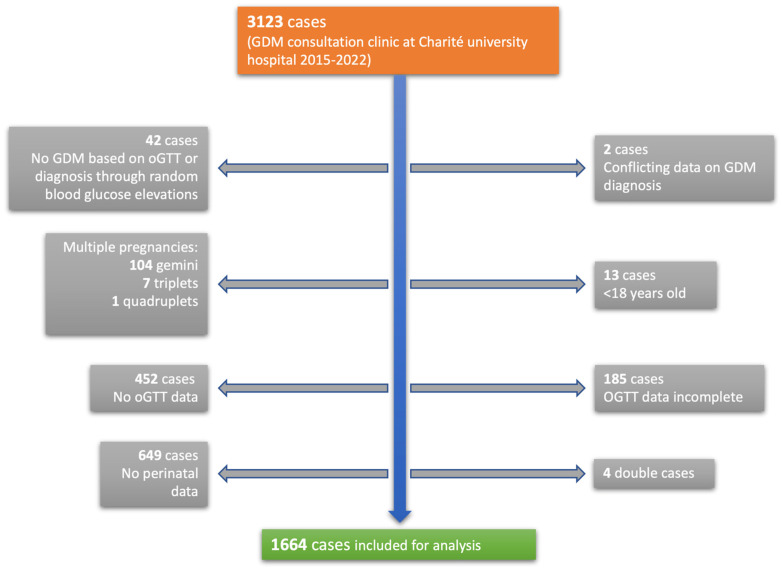
Obstetric data from 3123 pregnant women were collected and analyzed anonymously. A total of 1664 cases were eligible for analysis.

Obstetric data from 3123 pregnant women visiting an expert gestational diabetes consultation clinic at Charité University Hospital, from January 2015 to September 2022, were collected and analyzed anonymously. The Charité University Hospital is a tertiary perinatal center in the metropole region of Berlin, Germany. A total of 1664 patients were eligible for the analysis (Figure 2). The patients were screened and diagnosed through the 75 g oGTT and in case of pathological results were referred to our consultation clinic. Inclusion criteria were women ≥18 years with singleton pregnancies who were screened prior with a pathological glucose response in the 75 g oGTT and were subsequently diagnosed with GDM. The gestational week (GW) at the time of the pathological 75 g oGTT did not affect inclusion. High-risk patients who received early screening (before 24 GW) were included. Only women who delivered their babies at Charité University Hospital were eligible for the analysis. Exclusion criteria were multiple pregnancies, age < 18 years, missing or incomplete oGTT data, missing perinatal data and inconclusive documentation of GDM diagnosis. The oGTT data were considered incomplete if at least one glucose measurement of the three-point 75 g oGTT was missing and subsequently the categorization into one of the subtypes was not possible. Women who were diagnosed through random elevated blood glucose levels were excluded as well. Patients were categorized into three different groups using the three blood glucose values from the 75 g oGTT. Thresholds for pathological glucose levels were ≥92 mg/dL (5.1 mmol/L) fasting, ≥180 mg/dL (10 mmol/L) one hour after glucose application and ≥153 mg/dL (8.5 mmol/L) two hours after, according to IADPSG criteria [16]. An elevation in fasting glucose only, which was measured immediately before the application of the glucose solution, was considered isolated fasting hyperglycemia (GDM-IFH). If just one or both postprandial glucose values were elevated, this was considered isolated post-load hyperglycemia (GDM-IPH) and an elevation in fasting glucose and at least one of the postprandial glucose values was categorized as combined hyperglycemia (GDM-CH). The primary aim of the analysis was to assess the likelihood of delivering via cesarean section based on the glucose values of the 75 g oGTT. The secondary objective was to analyze maternal and fetal outcome parameters, such as vaginal operative birth, shoulder dystocia, perineal tear grade 3° and the need for episiotomy, blood loss and postpartum bleeding, and pre-eclampsia. Fetal outcomes included gestational age at delivery, birth weight and percentiles, fetal growth abnormalities such as intrauterine growth retardation (IUGR), small for gestational age (SGA), LGA (defined as growth ≥ 95th percentile) and low fetal weight (defined as growth < 30th percentile), premature delivery before 37 GW, intrauterine fetal demise (IUFD) and the need for intensive neonatal care admission after delivery. Cord pH levels and base excess were evaluated.

An analysis of the study population’s underlying characteristics was conducted regarding maternal age, gravidity and parity, pre-conceptional BMI and the previous diagnostic process including GW at first presentation, the 50 g oGCT and the 75 g oGTT. Gravidity and parity were assessed as continuous as well as categorical variables (gravida 1, 2 and ≥3; nullipara vs. ≥primipara and para 0, 1, 2, ≥3). 

This study received ethical approval from the ethics committee of Charité University Hospital on 6 February 2023 (EA2/255/22).

Data analysis was performed using SPSS Statistics by IBM (version 28.0.1.0) (Armonk, New York, United States of America). Categorical variables were compared among the subgroups using chi-square-tests and binomial logistic regression, and numbers and percentages were reported. Binomial logistic regression was conducted for outcome variables which showed significant differences among the subgroups and included subtype (categorical), pre-conceptional BMI (<18.5 = underweight, 18.5–23.9 = normal weight, 24–27.9 = overweight, 28–31.9 = obese, ≥32 = severely obese), age as a continuous variable and parity (nullipara vs. ≥primipara) as co-variates. Odds ratios and 95% confidence intervals were calculated and reported. Metric variables were compared using one-way ANOVA with subsequent post-hoc-analysis (Tukey and Games–Howell) and mean and standard deviation (SD) were reported. Results were considered statistically significant if the *p*-value was <0.05.

## 3. Results

### 3.1. Baseline Characteristics

**Figure 3:**
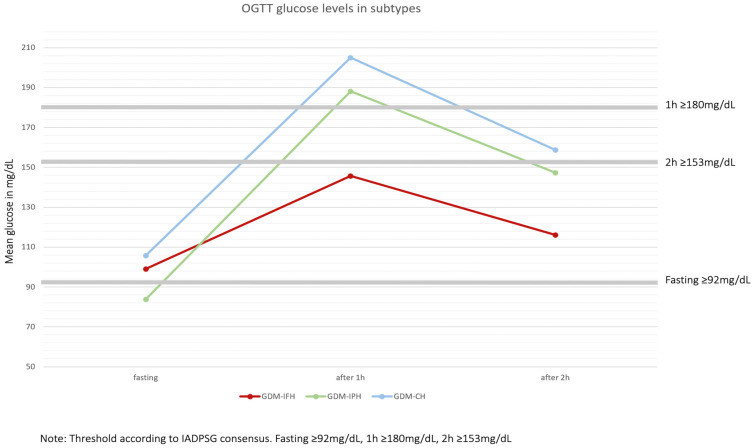
Attributes of each subtype: Isolated fasting hyperglycemia (GDM-IFH), isolated postprandial hyperglycemia (GDM-IPH), combined hyperglycemia (GDM-CH). Thresholds for pathological glucose levels were ≥92 mg/dL (5.1 mmol/L) fasting, ≥180 mg/dL (10 mmol/L) one hour after glucose application and ≥153 mg/dL (8.5 mmol/L) two hours after [2].

Of the 1664 patients, 553 were classified as GDM-IFH (33.2%), 418 as GDM-IPH (25.1%) and 693 as GDM-CH (41.6%) (Figure 1). Mothers from the GDM-IFH group were, on average, significantly younger (IFH: 31.89, IPH: 32.62, CH: 32.70, *p* = 0.024). Mean gravidity (IFH: 2.88, IPH: 2.92, CH: 3.32, *p* < 0.001) and parity (IFH: 1.31, IPH: 1.26, CH: 1.69, *p* < 0.001) were significantly higher in the GDM-CH group. The analysis of gravidity and parity as categorical variables revealed that among all subtypes, multigravidity (gravida ≥ 3: IFH: 48.3%, IPH: 46.9%, CH: 59.8%, *p* < 0.001) was significantly more common than gravida 1 (IFH: 22.8%, IPH: 27.8%, CH: 17.8%) or 2 (IFH: 28.9%, IPH: 25.4%, CH: 22.4%). The rate of nulliparous women was significantly higher in the GDM-IPH group (IFH: 33.8%, IPH: 39.7%, CH: 25.0%, *p* < 0.001). GDM-CH women had the highest rate of pluriparity (para ≥ 3: IFH: 15.6%, IPH: 15.6%, CH: 26.2%). Primiparity and biparity were similarly common when comparing the subtypes (IFH: 32.2%, IPH: 26.6%, CH: 27.8% and IFH: 18.4%, IPH: 18.2%, CH: 21.0%, respectively). The pre-conceptional BMI differed significantly between all the subgroups: GDM-IPH women displayed the lowest mean BMI and GDM-CH women the highest (IFH: 28.63, IPH: 26.10, CH: 29.69, *p* < 0.001). The 50 g oGCT was performed on average at 26 GW, with no significant difference between the groups (IFH: 25.62 GW, IPH: 25.89 GW, CH: 25.89 GW, *p* = 0.362). The GDM-IFH group was less likely to receive the 50 g oGCT before the 75 g oGTT than the other subgroups (IFH: 43.9%, IPH: 60.3%, CH: 59.2%, *p* < 0.001) and less likely to show a pathological glucose response in the test (IFH: 85.7%, IPH: 93.4%, CH: 93.9%, *p* < 0.001). Mean glucose levels in the 50 g oGCT were 144.75 mg/dL (8.03 mmol/L) in the GDM-IFH, 157.69 mg/dL (8.75 mmol/L) in the GDM-IPH and 166.37 mg/dL (9.23 mmol/L) in the GDM-CH group (*p* < 0.001). Women with GDM-IFH received the 75 g oGTT earlier than women in the GDM-IPH or GDM-CH groups (mean: IFH: 25.78 GW, IPH: 27.23 GW, CH: 26.53 GW, *p* < 0.001). In the GDM-IFH and GDM-CH groups the 75 g oGTT was performed before 24 GW more often than in the GDM-IPH group (13.2%, 11.8% vs. 7.2%; *p* = 0.009). Mean 75 g oGTT glucose levels before glucose intake, 1 h after and 2 h after, were 99.02 mg/dL (5.50 mmol/L), 145.63 mg/dL (8.08 mmol/L) and 116.16 mg/dL (6.45 mmol/L) in women with GDM-IFH, 83.77 mg/dL (4.65 mmol/L), 188.11 mg/dL (10.44 mmol/L) and 147.28 mg/dL (8.17 mmol/L) in women with GDM-IPH and 105.84 mg/dL (5.87 mmol/L), 204.98 mg/dL (11.38 mmol/L) and 158.80 mg/dL (8.81 mmol/L) in women with GDM-CH, respectively, (Figure 3). The mean initial presentation at the GDM consultation clinic was at 31 GW (IFH: 30.22 GW, IPH: 30.72 GW, CH: 30.39 GW, *p* = 0.270). A complete overview of the results is provided in Table 1. 

### 3.2. Maternal Outcome 

As provided in Table 2, the GDM-IFH group displayed the highest mean weight gain (IFH: 12.17 kg, IPH: 11.24 kg, CH: 10.90 kg, *p* = 0.008). GDM-IFH (17.5%, OR 1.946 [1.277–2.967], *p* = 0.002) as well as GDM-CH women were more likely to require insulin therapy (34.2%, OR 4.317 [2.915–6.392], *p* < 0.001) compared to GDM-IPH women (8.9%). GDM-CH patients had a higher likelihood of receiving insulin therapy (OR 2.218 [1.674–2.937], *p* < 0.001) compared to GDM-IFH. The use of long-acting insulin only was significantly more common in the GDM-IFH and GDM-CH groups (IFH: 13.0%, IPH: 5.3%, CH: 19.5%, *p* < 0.001) and the rate of combined insulin therapy was higher in the GDM-CH group (IFH: 3.8%, IPH: 1.4%, CH: 12.8%, *p* < 0.001). Two patients received treatment with metformin in combination with insulin.

The analysis showed no significant difference between rates of CS (primary and emergent, *p* = 0.811). However, there was a significant difference on primary and emergent CS specifically. GDM-IFH women were most likely to deliver via planned primary CS compared to GDM-IPH (IFH: 24.8%, IPH: 18.2%, CH: 21.5%, *p* = 0.047). The odds of delivering via primary CS were significantly increased only in GDM-IFH women (OR 1.376 [1.042–1.815], *p* = 0.024) vs. GDM-CH patients. Women categorized as GDM-IPH (23.9%, OR 1.643 [1.173–2.302], *p* = 0.004) or GDM-CH (21.7%, OR 1.48 [1.094–2.003], *p* = 0.011) were at higher risk of an emergent CS compared to GDM-IFH women (16.6%). The rate of vaginal operative delivery differed significantly with 10% in GDM-IPH patients vs. 5.5% in GDM-CH patients (IFH: 8.1%, *p* = 0.016). There were no significant differences concerning the rates of shoulder dystocia (IFH: 1.2%, IPH: 0.0%, CH: 2.0%, *p* = 0.081), episiotomy (IFH: 8.3%, IPH: 11.6%, CH: 8.9%, *p* = 0.389), third degree perineal tear (IFH: 1.9%, IPH: 1.2%, CH: 1.0%, *p* = 0.620), pre-eclampsia (IFH: 3.3%, IPH: 1.7%, CH: 3.8%, *p* = 0.143) and HELLP syndrome (IFH: 0.4%, IPH: 0.2%, CH: 0.1%, *p* = 0.739). The analysis of blood loss as a continuous variable showed no significant difference (*p* = 0.643), as well as the incidence of blood loss ≥ 1000 mL (*p* = 0.867) and ≥1500 mL (*p* = 0.400). The risk of postpartum bleeding was similar throughout the subgroups (*p* = 0.893).

### 3.3. Fetal Outcome 

The distribution of the fetus’s sex did not differ significantly between the groups (*p* = 0.760). The mean gestational age at delivery was slightly higher in the GDM-IFH group than in the GDM-CH group (IFH: 39.70 GW, IPH: 39.51 GW, CH: 39.41 GW, *p* = 0.008). The rates of premature delivery before 37 GW were similar among the subtypes (*p* = 0.054). APGAR scores at 1 min (*p* = 0.088), 5 min (*p* = 0.110) and 10 min after delivery (*p* = 0.061), arterial cord pH values (*p* = 0.446) and base excess (*p* = 0.906) did not differ significantly among the subgroups. Neonates of GDM-IFH and GDM-CH mothers displayed a significantly higher mean birthweight (IFH: 3470.71 g, IPH: 3327.59 g, CH: 3460.21 g, *p* < 0.001) as well as birth weight percentiles (IFH: 54.68, IPH: 49.43, CH: 57.51, *p* < 0.001) compared to neonates of GDM-IPH women. GDM-IFH (12.3%, OR 1.657 [1.020–2.692], *p* = 0.041) and GDM-CH women (13.5%, OR 1.671 [1.046–2.668], *p* = 0.032) were at higher risk of delivering neonates that were LGA compared to GDM-IPH (6.5%). The SGA rate was significantly higher among GDM-IPH women (IFH: 7.6%, IPH: 11.5%, CH: 7.1%, *p* = 0.027). In the logistic regression analysis, SGA did not reach statistical significance. However, GDM-IPH women (30.7%, OR 1.379 [1.029–1.847], *p* = 0.031) displayed an association with low fetal weight (<30th percentile) in comparison to GDM-CH women (21.6%, IFH: 26.3%). There was no significant difference in neonatal intensive care admission (*p* = 0.086) and the rate of IUFD (*p* = 0.104) (Table 3).

### 3.4. Effects of Covariates on Fetomaternal Outcome

In the logistic regression model, not only the three 75 g oGTT subtypes were associated with fetomaternal outcome. In particular, parity and pre-conceptional BMI seemed to significantly affect the perinatal outcomes. Nulliparous women displayed smaller odds of primary CS (OR 0.435 [0.320–0.593], *p* < 0.001), but were at higher risk of delivering via emergent CS (OR 3.534 [2.700–4.625], *p* < 0.001). Their likelihood of operative vaginal delivery was higher as well (OR 6.353 [4.100–9.843], *p* < 0.001). Nulliparity increased the odds of delivering offspring that had low fetal weight (OR 1.583 [1.238–2.025], *p* < 0.001) or was SGA (OR 2.155 [1.484–3.129], *p* < 0.001), while it reduced the odds of delivering LGA neonates (OR 0.548 [0.365–0.823], *p* = 0.004). Pre-conceptional BMI was associated with emergent CS (*p* = 0.001), vaginal operative delivery (*p* = 0.007) and LGA (*p* < 0.001). A BMI categorized as overweight, obese or severely obese increased the likelihood of emergent CS (OR 1.451 [1.011–2.083], 1.585 [1.070–2.349] and 2.019 [1.385–2.941], respectively) and delivering an LGA fetus (OR 2.020 [1.145–3.565], 2.112 [1.172–3.805] and 3.639 [2.110–6.276], respectively). Underweight mothers (OR 2.911 [1.148–7.382]) were at significantly higher risk of delivering via vaginal operative delivery. Severely obese women (OR 0.425 [0.217–0.832]) on the other hand were at lower risk compared to normal weight women. Maternal age was associated with primary (OR 1.042 [1.019–1.067], *p* < 0.001 for the increase of 1 year) as well as emergent CS (OR 1.031 [1.007–1.055], *p* = 0.010 for the increase of 1 year). 

## 4. Discussion

**Figure 4:**
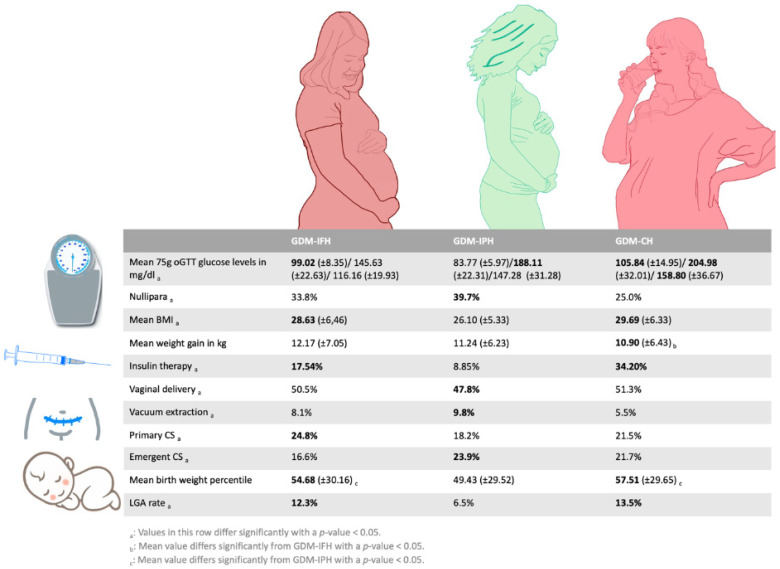
An overview of significant differences between women of the three subtypes regarding parity, BMI and weight gain, insulin therapy, mode of delivery and fetal growth.

Our analysis was able to corroborate the existence of three different types of metabolic phenotypes in women with GDM based on the 75 g oGTT levels. Each group revealed specific associations regarding the baseline characteristics as well as fetomaternal outcomes (Figure 4).

GDM-IFH women were the youngest but had the highest mean weight gain. GDM-CH women were the oldest and displayed the highest number of previous deliveries. Both groups presented with significantly higher pre-conceptional BMI than the GDM-IPH group.

GDM-IFH and GDM-CH women displayed an overall higher rate of well-established GDM risk factors, such as higher maternal age [17] or BMI [18]. This may explain, why GDM-IFH and GDM-CH women received the 75 g oGTT more frequently before 24 GW. The rate of a previous GDM was not assessable through our dataset, although it is an important contributing factor for the development of GDM and should be investigated in further studies [19].

Our results revealed a strong association of GDM-IFH and GDM-CH with the requirement of insulin therapy, which is corroborated by recent studies [14,20,21,22,23]. Kotzaeridi et al. affirmed a “worse metabolic profile”, higher BMI and an increased requirement for glucose-lowering medications in women with elevated fasting glucose, especially GDM-CH women [15]. Their study additionally revealed a significantly higher BMI in GDM-IFH and GDM-CH patients compared to women without glucose intolerance.

It is well-established that GDM in itself increases the risk of neonates being LGA [4]. We found that GDM-IFH and GDM-CH mothers delivered offspring with significantly higher birth weight as well as birth weight percentiles and increased odds of being LGA at the time of delivery compared to the GDM-IPH group. This aligns with findings of numerous studies, which previously demonstrated that maternal fasting glucose is strongly associated with LGA and higher birth weight [5,12,13]. An analysis by Black et al. revealed higher rates of LGA and increased birth weight in women with fasting hyperglycemia, not only compared to patients with post-load hyperglycemia but to non-GDM patients as well [13]. Uvena-Celebrezze et al. were able to establish a correlation of maternal fasting glucose and neonatal fat mass in a study that used the self-monitoring of glucose levels [24]. Zawiejska et al. showed that maternal fasting hyperglycemia was associated with birthweight ≥ 4000 g [14].

The higher rate of LGA in GDM-IFH and GDM-CH groups possibly contributes to an increased rate of primary CS in these groups. The rates of primary CS were highest in the GDM-IFH group, however in the binomial logistic regression only the association of GDM-IFH with primary CS in comparison to GDM-CH reached statistical significance. 

The risk of emergent CS was significantly increased in the GDM-IPH and GDM-CH groups compared to GDM-IFH, whereas we found no significant difference among the subgroups regarding cesarean section in general.

GDM-IPH women presented with a lower pre-conceptional BMI, were more likely nulliparous and required insulin therapy less often. 

While we found a higher rate of vaginal operative deliveries in women with GDM-IPH, there was no association of the subtypes and vaginal operative deliveries in the logistic regression analysis. However, the odds of a vaginal operative delivery were significantly increased if women were underweight and/or nulliparous. These characteristics were more frequently displayed in the GDM-IPH group; therefore, this could explain the higher rate of vaginal operative delivery. This goes along with a recent analysis, that was able to demonstrate an association of vacuum extraction and nulliparity [25]. Ramos et al. examined women requiring operative delivery assistance and found a decreased likelihood of vaginal operative delivery in women with pre-pregnancy obesity [26].

The rate of SGA was significantly higher in women of the GDM-IPH group; however, SGA did not reach statistical significance in the logistic regression. This may be explained by the strong association between nulliparity and SGA we found in our analysis, as well as the significantly higher rate of nulliparous women in the GDM-IPH group. 

Previous studies found correlations of post-load hyperglycemia and gestational hypertension, hyperbilirubinemia and preterm delivery, whereas preterm delivery did not differ significantly among our subgroups and gestational hypertension and hyperbilirubinemia were not evaluated in this study [12,13,14].

Of note, IUFD and shoulder dystocia did not reach statistical significance, but all reported cases in our sample occurred either in women of the GDM-IFH or GDM-CH subgroups. Several other studies have previously shown associations of maternal fasting glucose and LGA or macrosomia with shoulder dystocia [27]. They found fetal macrosomia to be a mediating factor between maternal fasting hyperglycemia and shoulder dystocia. A meta-analysis by Farrar et al. showed associations of fasting as well as post-load glucose levels with shoulder dystocia, although an increase in fasting glucose was more strongly associated [5].

A limitation of this study was the lack of healthy controls, as we collected the data solely from the gestational diabetes consultation without a control group. The inclusion of normal glucose tolerant women in previous studies, such as Kotzaeridi et al., has provided further insight and the advantage of contextualizing different pathological glucose response patterns [15]. The study population consists of patients exclusively from one GDM consultation clinic in Berlin, Germany, which may have an impact on the generalizability of the results. Additionally, the sample sizes for the individual analyses of variables were not the same throughout the study, due to sporadically missing data. On the other hand, an important advantage is the large sample size and amount of different baseline and outcome parameters that were assessed in this study. 

## 5. Conclusions

To conclude, we did observe significant differences between the GDM subtypes regarding their underlying characteristics and the course of their diagnostic process and were able to identify subtypes that were at higher risk of certain adverse perinatal outcomes. Women categorized as GDM-IFH or GDM-CH were more likely to need a type of insulin therapy, displayed a higher BMI, and their offspring had higher birthweight, birth weight percentiles and were more likely a LGA fetus, while neonates of women with GDM-IPH were at increased risk of low fetal weight. GDM-IFH was associated with primary CS, while GDM-IPH and GDM-CH were associated with emergent CS. 

This analysis suggests that the categorization based on the 75 g oGTT glucose levels could be a practicable approach to adapt the prenatal care of women with GDM based on their risk factors. In the future, prospective studies taking the maternal risk factors into account should be conducted, possibly including interventions for women at risk.

## Figures and Tables

**Table 1:** Baseline characteristics. Baseline characteristics on maternal age, pregnancy history, BMI, glucose screening and screening results.

		Subtype											
		GDM-IFH (n = 553)				GDM-IPH (n = 418)				GDM-CH (n = 693)			
		Mean	SD	n	%	Mean	SD	n	%	Mean	SD	n	%
Maternal age		31.89_a_	5.78			32.62_b_	5.37			32.70_b_	5.40		
Gravida		2.88_a_	1.81			2.92_a_	2.06			3.32_b_	1.99		
Para		1.31_a_	1.44			1.26_a_	1.53			1.69_b_	1.52		
Parity	Nullipara			187_a_	33.8%			166_a_	39.7%			173_b_	25.0%
	≥Primipara			366_a_	66.2%			252_a_	60.3%			518_b_	75.0%
Preconceptional BMI		28, 63_a_	6,46			26, 10_b_	5,33			29, 69_c_	6, 33		
50 g oGCT	yes			243_a_	43.9%			252_b_	60.3%			410_b_	59.2%
	no			310_a_	56.1%			166_b_	39.7%			283_b_	40.8%
GW at 50 g oGCT		25, 62_a_	2.18			25, 89_a_	2.11			25, 85_a_	2.38		
Glucose 50 g oGCT in mg/dL		144, 75_a_	18.67			157, 69_b_	21.71			166, 37_c_	30.95		
50 g oGCT ≥ 135 mg/dL	yes			198_a_	85.7%			225_b_	93.4%			368_b_	93.9%
	no			33_a_	14.3%			16_b_	6.6%			24_b_	6.1%
GW at 75 g oGTT		25, 78_a_	4.67			27, 23_b_	3.76			26, 53_c_	4.27		
75 g oGTT	before GW 24+0			73_a_	13.2%			30_b_	7.2%			82_a_	11.8%
	after GW 24+0			480_a_	86.8%			388_b_	92.8%			611_a_	88.2%
Fasting glucose in mg/dL		99, 02_a_	8.35			83, 77_b_	5.97			105, 84_c_	14.95		
Glucose after 1 h in mg/dL		145, 63_a_	22.63			188, 11_b_	22.31			204, 98_c_	32.01		
Glucose after 2 h in mg/dL		116, 16_a_	19.93			147, 28_b_	31.28			158, 80_c_	36.67		
GW at first presentation		30, 22_a_	4.76			30, 72_a_	4.42			30, 39_a_	4.97		

Note: Values in the same row that are marked with different subscripts (a,b and c) differ significantly with a *p*-value < 0.05.

**Table 2:** Maternal outcome. Maternal outcome regarding GDM therapy, pregnancy complications and delivery mode and birth complications.

		Subtype											
		GDM-IFH (n = 553)				GDM-IPH (n = 418)				GDM-CH (n = 693)			
		Mean	SD	n	%	Mean	SD	n	%	Mean	SD	n	%
Weight gain		12.17_a_	7.05			11.24_b_	6.23			10.90_b_	6.43		
Insulin	long acting insulin only			72_a_	13.0%			22_b_	5.3%			135_c_	19.5%
	combined insulin			21_a_	3.8%			6_b_	1.4%			89_c_	12.8%
	short acting insulin only			4_a_	0.7%			9_a_	2.2%			13_a_	1.9%
	no			456_a_	82.5%			381_b_	91.1%			456_c_	65.8%
Metformin	yes			1_a_	0.2%			0^2^	0.0%			1_a_	0.1%
	no			552_a_	99.8%			418^2^	100.0%			692_a_	99.9%
Preeclampsia	yes			18_a_	3.3%			7_a_	1.7%			26_a_	3.8%
	no			535_a_	96.7%			411_a_	98.3%			667_a_	96.2%
HELLP	yes			2_a_	0.4%			1_a_	0.2%			1_a_	0.1%
	no			551_a_	99.6%			417_a_	99.8%			692_a_	99.9%
Delivery mode	Vaginal			279_a_	50.5%			200_a_	47.8%			355_a_	51.3%
	Vacuum extraction			45_a,b_	8.1%			41_a_	9.8%			38_b_	5.5%
	Primary CS			137_a_	24.8%			76_b_	18.2%			149_a,b_	21.5%
	Emergent CS			92_a_	16.6%			100_b_	23.9%			150_b_	21.7%
	Forceps extraction			0	0.0%			1	0.2%			0	0.0%
Shoulder dystocia	yes			4_a_	1.2%			0	0.0%			8_a_	2.0%
	no			320_a_	98.8%			242	100.0%			385_a_	98.0%
Perineal tear	yes			114_a_	35.2%			95_a_	39.3%			133_a_	33.8%
	no			210_a_	64.8%			147_a_	60.7%			260_a_	66.2%
Third degree perinealtear	yes			6_a_	1.9%			3_a_	1.2%			4_a_	1.0%
	no			318_a_	98.1%			239_a_	98.8%			389_a_	99.0%
Episiotomy	yes			27_a_	8.3%			28_a_	11.6%			35_a_	8.9%
	no			297_a_	91.7%			214_a_	88.4%			358_a_	91.1%
Blood loss in mL		443_a_	257			450_a_	373			433_a_	257		
Blood loss ≥1000 mL	yes			26_a_	4.8%			18_a_	4.5%			28_a_	4.2%
	no			516_a_	95.2%			385_a_	95.5%			645_a_	95.8%
Blood loss ≥1500 mL	yes			10_a_	1.8%			9_a_	2.2%			8_a_	1.2%
	no			532_a_	98.2%			394_a_	97.8%			665_a_	98.8%
Postpartum bleeding	yes			27_a_	4.9%			22_a_	5.3%			38_a_	5.5%
	no			526_a_	95.1%			396_a_	94.7%			655_a_	94.5%

Note: Values in the same row that are marked with different subscripts (a, b and c) differ significantly with a *p*-value < 0.05.

**Table 3:** Fetal outcome. Fetal outcome regarding gestational age at delivery, birthweight, Apgar and cord blood pH.

Subtype													
GDM-IFH (n = 553)						GDM-IPH (n = 418)				GDM-CH (n = 693)			
n			%	Mean	SD	n	%	Mean	SD	n	%	Mean	SD
Sex of fetus	male	305_a_	55.2%			234_a_	56.3%			372_a_	54.0%		
	female	248_a_	44.8%			182_a_	43.8%			317_a_	46.0%		
GW at delivery				39.70_a_	1.50			39.51_a,b_	2.08			39.41_b_	1.92
Prematurity <37 GW	yes	16_a_	2.9%			25_a_	6.0%			35_a_	5.1%		
	no	537_a_	97.1%			393_a_	94.0%			657_a_	94.9%		
Birthweight				3470.71_a_	556.88			3327.59_b_	609.55			3460.21_a_	605.72
Birthweight percentile				54.68_a_	30.16			49.43_b_	29.52			57.51_a_	29.65
IUGR	yes	9_a_	1.6%			13_a_	3.1%			9_a_	1.3%		
	no	543_a_	98.4%			404_a_	96.9%			682_a_	98.7%		
SGA	yes	42_a_	7.6%			48_b_	11.5%			49_a_	7.1%		
	no	510_a_	92.4%			369_b_	88.5%			642_a_	92.9%		
Low fetal weight	yes	145_a,b_	26.3%			128_a_	30.7%			149_b_	21.6%		
	no	407_a,b_	73.7%			289_a_	69.3%			542_b_	78.4%		
LGA	yes	68_a_	12.3%			27_b_	6.5%			93_a_	13.5%		
	no	484_a_	87.7%			390_b_	93.5%			598_a_	86.5%		
APGAR at 1 min				8.62_a_	1.20			8.64_a_	1.03			8.49_a_	1.38
APGAR at 5 min				9.56_a_	0.91			9.49_a_	0.96			9.43_a_	1.17
APGAR at 10 min				9.81_a_	0.66			9.75_a_	0.77			9.70_a_	1.01
Cord pH				7.24_a_	0.07			7.24_a_	0.07			7.24_a_	.07
Base excess				−4.44_a_	2.96			−4.38_a_	3.25			−4.36_a_	3.24
NICU	yes	28_a_	5.1%			36_a_	8.6%			45_a_	6.5%		
	no	525_a_	94.9%			382_a_	91.4%			648_a_	93.5%		
IUFD	yes	1_a_	0.2%			0	0.0%			5_a_	0.7%		
	no	552_a_	99.8%			418	100.0%			687_a_	99.3%		

Values in the same row that are marked with different subscripts (a, b and c) differ significantly with a *p*-value < 0.05.

## Data Availability

The data presented in this study are available on request from the corresponding author.

## Abbreviations

GDM	gestational diabetes mellitus
oGTT	oral glucose tolerance test
oGCT	oral glucose challenge test
CS	cesarean section
GDM-IFH	gestational diabetes with isolated fasting hyperglycemia
GDM-IPH	gestational diabetes with isolated postprandial hyperglycemia
GDM-CH	gestational diabetes with combined hyperglycemia
LGA	large for gestational age
IUGR	intrauterine growth retardation
SGA	small for gestational age
GW	gestational week
IUFD	intrauterine fetal death
